# A cardiovascular magnetic resonance imaging-based pilot study to assess coronary microvascular disease in COVID-19 patients

**DOI:** 10.1038/s41598-021-95277-z

**Published:** 2021-08-02

**Authors:** Stefanos Drakos, Grigorios Chatzantonis, Michael Bietenbeck, Georg Evers, Arik Bernard Schulze, Michael Mohr, Helena Fonfara, Claudia Meier, Ali Yilmaz

**Affiliations:** 1 Department of Cardiology I, University Hospital Münster, Albert-Schweitzer-Campus 1, Building A1, 48149 Münster, Germany; 2 Department of Medicine A, Hematology, Oncology and Pulmonary Medicine, University Hospital Münster, Münster, Germany

**Keywords:** Cardiology, Infection

## Abstract

Coronavirus disease 2019 (COVID-19) is caused by severe acute respiratory syndrome coronavirus-2 (SARS-CoV-2) and is primarily characterised by a respiratory disease. However, SARS-CoV-2 can directly infect vascular endothelium and subsequently cause vascular inflammation, atherosclerotic plaque instability and thereby result in both endothelial dysfunction and myocardial inflammation/infarction. Interestingly, up to 50% of patients suffer from persistent exercise dyspnoea and a post-viral fatigue syndrome (PVFS) after having overcome an acute COVID-19 infection. In the present study, we assessed the presence of coronary microvascular disease (CMD) by cardiovascular magnetic resonance (CMR) in post-COVID-19 patients still suffering from exercise dyspnoea and PVFS. N = 22 patients who recently recovered from COVID-19, N = 16 patients with classic hypertrophic cardiomyopathy (HCM) and N = 17 healthy control patients without relevant cardiac disease underwent dedicated vasodilator-stress CMR studies on a 1.5-T MR scanner. The CMR protocol comprised cine and late-gadolinium-enhancement (LGE) imaging as well as velocity-encoded (VENC) phase-contrast imaging of the coronary sinus flow (CSF) at rest and during pharmacological stress (maximal vasodilation induced by 400 µg IV regadenoson). Using CSF measurements at rest and during stress, global myocardial perfusion reserve (MPR) was calculated. There was no difference in left ventricular ejection-fraction (LV-EF) between COVID-19 patients and controls (60% [57–63%] vs. 63% [60–66%], p = NS). There were only N = 4 COVID-19 patients (18%) showing a non-ischemic pattern of LGE. VENC-based flow measurements showed that CSF at rest was higher in COVID-19 patients compared to controls (1.78 ml/min [1.19–2.23 ml/min] vs. 1.14 ml/min [0.91–1.32 ml/min], p = 0.048). In contrast, CSF during stress was lower in COVID-19 patients compared to controls (3.33 ml/min [2.76–4.20 ml/min] vs. 5.32 ml/min [3.66–5.52 ml/min], p = 0.05). A significantly reduced MPR was calculated in COVID-19 patients compared to healthy controls (2.73 [2.10–4.15–11] vs. 4.82 [3.70–6.68], p = 0.005). No significant differences regarding MPR were detected between COVID-19 patients and HCM patients. In post-COVID-19 patients with persistent exertional dyspnoea and PVFS, a significantly reduced MPR suggestive of CMD—similar to HCM patients—was observed in the present study. A reduction in MPR can be caused by preceding SARS-CoV-2-associated direct as well as secondary triggered mechanisms leading to diffuse CMD, and may explain ongoing symptoms of exercise dyspnoea and PVFS in some patients after COVID-19 infection.

## Introduction

Coronavirus disease 2019 (COVID-19) is caused by severe acute respiratory syndrome coronavirus-2 (SARS-CoV-2) and is primarily characterised by a respiratory disease. However, SARS-CoV-2 can directly infect vascular endothelium via angiotensin-converting enzyme 2 (ACE2) receptor and subsequently cause vascular inflammation, atherosclerotic plaque instability and thereby result in both myocardial inflammation and infarction^1,2^. In addition to such a direct endothelial injury, SARS-CoV-2 may also trigger secondary (auto-)immune responses and cause a massive proinflammatory cytokine release, thereby further aggravating endothelial injury and coronary microvascular disease (CMD)^3^.

Under normal conditions, there is an endothelium-dependent dilation or constriction of coronary arterioles in response to surrounding myocardial metabolic and/or inflammatory conditions to match myocardial oxygen demand and supply. Dysfunction of the coronary microvasculature may occur as a consequence of disturbances in the complex signalling pathways in endothelial as well as smooth muscle cells, but also as a consequence of abnormal cytokine release and/or thrombotic microvascular obstruction—caused by e.g. viral infections^4^. Noteworthy, so far there are no data regarding the presence and severity of CMD in patients with active COVID-19 infection or in those who recently recovered from COVID-19. Interestingly, up to 50% of patients suffer from persistent exercise dyspnoea and a post-viral fatigue syndrome (PVFS) after having overcome an acute COVID-19 infection^5^. Moreover, recent autopsy data showed the presence of SARS-CoV-2 transcriptional activity in human cardiomyocytes in COVID-19 patients who died due to respiratory failure in the absence of clinical signs of acute cardiac involvement^6^.

So far, there are no non-invasive imaging modalities that allow to directly visualize the human coronary microvasculature. Therefore, indirect approaches such as measurement of myocardial perfusion reserve (MPR) are used to assess the presence and extent of CMD. A rather simple, however, appropriate approach to determine global MPR is based on velocity-encoded (VENC) phase-contrast imaging of coronary sinus flow (CSF) during rest and stress conditions by cardiovascular magnetic resonance (CMR) imaging. In the past, CSF measurements obtained by CMR were compared to positron-emission-tomography (PET) results and showed an excellent agreement between these methods^7^. Moreover, the diagnostic performance and benefit of VENC-based CSF measurements were already addressed in different clinical settings^8–12^. Hence, we assessed the presence of CMD by CMR-based CSF measurements in post-COVID-19 patients still suffering from exercise dyspnoea and PVFS in the present hypothesis-generating pilot-study.

## Methods

### Study population

All patients included in this study underwent a vasodilator stress CMR examination. The first study group (COVID-19 group) comprised N = 22 patients with previous positive testing for COVID-19 (reverse transcriptase-polymerase chain reaction) between April and October 2020 and after 1–6 months of recovery from SARS-CoV-2 infection. Only those COVID-19 patients with persisting symptoms of exertional dyspnoea (NYHA class II or III) and presence of fatigue suggestive of post-viral fatigue syndrome (PVFS) were included. We did not include severely diseased COVID-19 patients with critical illness and/or acute respiratory distress syndrome (ARDS) during the acute phase and enrolled only those post-COVID-19 patients who did not require mechanical ventilatory or catecholaminergic support during the acute phase in order to minimise potential confounding effects caused by chronic pulmonary disease. Moreover, ongoing relevant pulmonary disease was excluded by either spirometry, chest X-ray or computed tomography of the thorax prior to the CMR study. The second study group (HCM group) comprised N = 16 patients with classical non-obstructive hypertrophic cardiomyopathy (HCM) showing preserved left ventricular ejection fraction (LV-EF) ≥ 50% and LV wall thickness ≥ 15 mm that could not be explained by abnormal loading conditions. Patients with any history of relevant coronary artery disease (CAD), congenital heart disease and in particular persistent left superior vena cava were excluded. Furthermore, a control group (N = 17) without any structural and functional cardiac abnormalities and a low pre-test probability of CAD was retrospectively recruited. The local ethics committee (Ethikkommission der Ärztekammer Westalen-Lippe und der Westfälischen Wilhelms-Universität) approved the study protocol and written informed consent was obtained from every patient prior to the CMR study. All methods were carried out in accordance with relevant guidelines and regulations.

### CMR acquisition

As summarized previously^8^, CMR imaging was performed on a 1.5-T system (Ingenia or Ambition, Philips Healthcare, Best, The Netherlands) during breath-hold and with ECG-triggering. The CMR protocol comprised cine-imaging, myocardial stress-perfusion and late-gadolinium-enhancement (LGE)-imaging, approximately 10–15 min after a cumulative gadolinium (Gadobutrol) dose of 0.15 mmol/kg. In addition, through-plane velocity-encoded (VENC) coronary sinus (CS) flow measurements with a pre-defined VENC factor of 100 cm/s (reflecting the maximum resolvable flow velocity and with adoption as needed) were performed at rest and approximately 1 min after regadenoson administration at maximal vasodilation. The imaging plane was carefully planned perpendicular to the CS on two orthogonal views once the CS was located in the atrioventricular groove in a stack of axial images. To measure the maximum of venous drainage, the flow measurement slice was positioned closely to the CS orifice in the right atrium. Stress and rest imaging were timed 10–15 min apart.

### CMR data analysis

As illustrated previously^8,12^, image analysis and interpretation were performed using commercially available software (cvi42, Circle Cardiovascular Imaging, Calgary, Alberta, Canada) by two experienced physicians regarding CMR analysis. Ventricular volumes and LV mass were determined by contouring short-axis cine images whereas LGE images were visually assessed. Coronary sinus flow (CSF) measurements were performed through manual tracing of the CS on the phase-contrast magnitude images throughout the whole cardiac cycle. For the measurement of global myocardial perfusion reserve (MPR) per beat, the absolute volumetric measurement of the CSF during stress was divided with the absolute volumetric measurement of the CSF at rest. In order to adequately consider potential heart-rate (HR)-associated differences, MPR per minute was also calculated by multiplying stress-CSF and rest-CSF values with their respective HR before calculating their ratio.

### Statistical analysis

Statistical analysis was performed with SPSS (version 26.0, IBM Corp., Armonk, NY). All continuous variables showed non-normal distribution and are expressed as median (plus 25th and 75th percentile) values. Categorical variables are expressed as frequency with percentage. Kruskal–Wallis test was used for the comparison of all continuous (non-normally distributed) variables Mann–Whitney-*U* test was used in case of comparison of two groups. For the comparison of categorical variables, the Chi-square test with Bonferroni correction was used. A p-value < 0.05 was considered statistically significant.

### Ethics approval and consent to participate

The study protocol complies with the Declaration of Helsinki. Written informed consent was obtained from every patient.

## Results

### Study population

The clinical characteristics and comorbidities of the study population are summarized in Table 1. The HCM group was significantly older than the COVID-19 group (p = 0.014), whereas the COVID-19 group was significantly older than the control group (p = 0.001). Regarding cardiovascular risk factors, only arterial hypertension showed a significantly higher prevalence in the HCM group compared to the COVID-19 group (81% vs 14%, p < 0.001). No other significant differences were observed between the groups.

**Table 1:** Patient characteristics.

	COVID-19	HCM	Control	p-value (COVID-19 vs. HCM)	p-value (COVID-19 vs. Control)
	N = 22	N = 16	N = 17		
Male, n (%)	14 (64)	13 (81)	8 (47)	0.30	0.35
Age, years	51 (45–59)	71 (61–78)	39 (25–42)	**0.014**	**0.001**
BMI, kg/m^2^	26 (24–29)	28 (25–29)	27 (22–32)	0.32	0.70
Hypertension, n (%)	3 (14)	13 (81)	4 (24)	**< 0.001**	0.68
Diabetes, n (%)	0 (0)	4 (25)	0 (0)	0.05	1.00
High cholesterol, n (%)	3 (14)	1 (6)	2 (12)	0.62	1.00
Current smoker, n (%)	3 (14)	2 (13)	1 (6)	1.00	0.62

Bold values indicates p-value < 0.05.

### Conventional CMR findings

**Figure 1:**
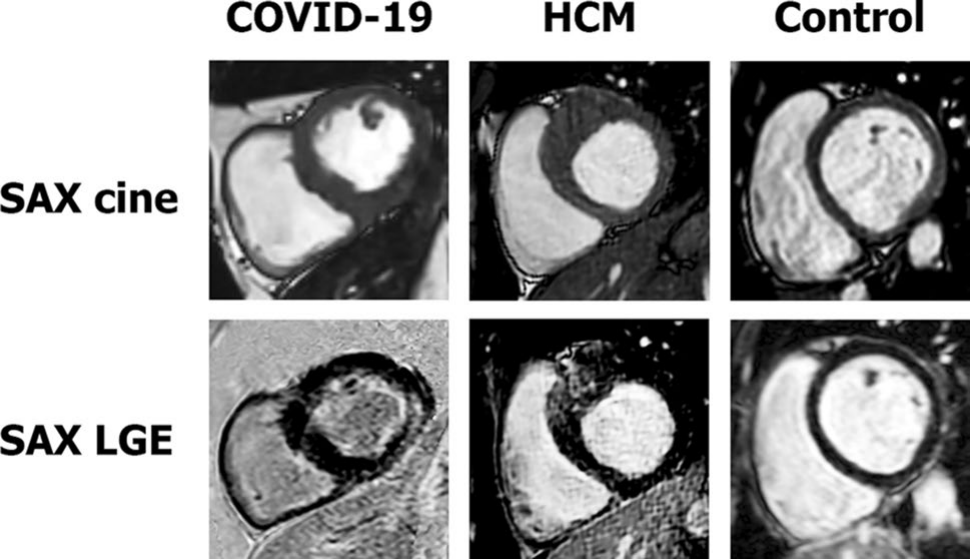
Cardiovascular magnetic resonance (CMR) cine-images (1st row) and late gadolinium enhancement (LGE)-images (2nd row) in short-axis views of patients who recently recovered from coronavirus disease (COVID-19) (1st column), with hypertrophic cardiomyopathy (HCM) (2nd column) and a healthy control (3rd column).

All anatomic, functional and structural CMR findings are shown in Table 2. There was no significant difference in left ventricular ejection fraction (LV-EF) between the three groups (60 [57–63] % in COVID-19 vs. 65 [58–71] % in HCM (p = 0.08) vs. 63 [60–66] % in controls (p = 0.07)). As expected, LV mass index and maximal LV wall thickness were significantly lower in the COVID-19 group compared to the HCM group (51 [45–62] vs. 70 [61–75] g/m^2^; 10 [8–11] mm vs. 16 [15–17] mm, both p < 0.001). The presence and extent of LGE were significantly more frequent/higher in the HCM group compared to the COVID-19 group (both p < 0.001). In particular, a non-ischemic, predominantly subepicardial pattern of LGE was detected in the basal LV segments in 4 (18%) COVID-19 patients whereas a rather diffuse septal pattern was observed in hypertrophic segments in 13 (81%) HCM patients. Representative examples of LGE patterns are illustrated in Fig. 1.

**Table 2:** Conventional CMR parameters.

	COVID-19	HCM	Control	p-value (COVID-19 vs. HCM)	p-value (COVID-19 vs. Control)
	N = 22	N = 16	N = 17		
LV-EF, %	60 (57–63)	65 (58–71)	63 (60–66)	0.08	0.07
LV-EDV index, ml/m^2^	78 (63–90)	67 (62–73)	84 (78–91)	0.13	0.20
LV-ESV index, ml/m^2^	33 (23–37)	24 (19–28)	32 (26–35)	**0.008**	1.00
LV mass index, g/m^2^	51 (45–62)	70 (61–75)	58 (49–62)	**< 0.001**	0.54
Max. LV wall thickness, mm	10 (8–11)	16 (15–17)	9 (7–11)	**< 0.001**	1.00
RV-EF, %	58 (52–60)	63 (57–65)	64 (59–66)	0.15	**0.005**
RV-EDV index, ml/m^2^	80 (66–88)	66 (61–73)	86 (76–94)	**0.041**	0.43
RV-ESV index, ml/m^2^	33 (25–43)	24 (22–29)	29 (25–39)	**0.028**	1.00
LGE presence, n (%)	4 (18)	13 (81)	0 (0)	**< 0.001**	0.19
LGE extent, %	0 (0–0)	3 (1–6)	0 (0–0)	**< 0.001**	0.81

Bold values indicates p-value < 0.05.

### Coronary sinus flow (CSF) and global myocardial perfusion reserve (MPR) findings

**Figure 2:**
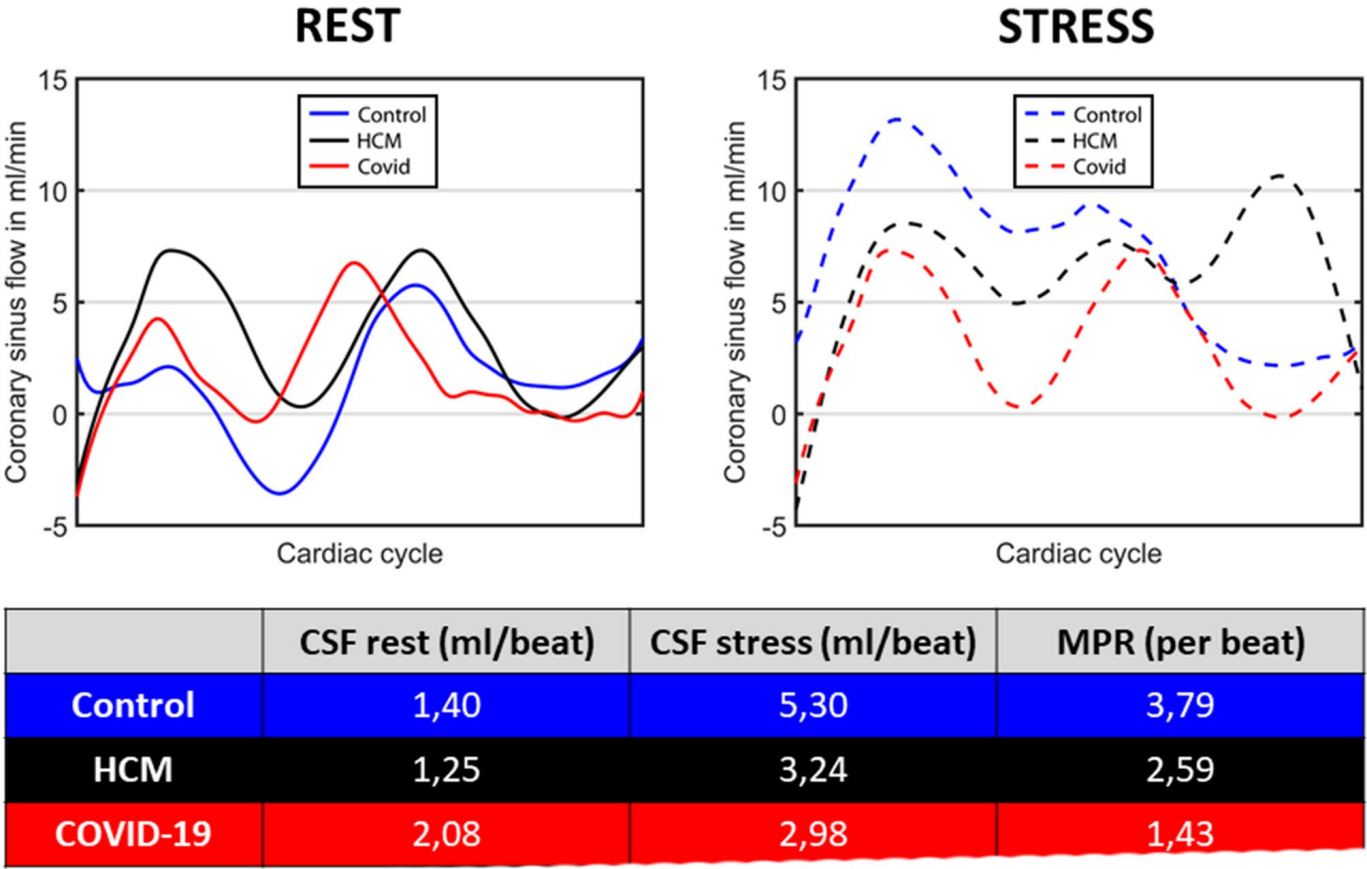
Flow-velocity curves of the coronary sinus (CS) of one representative patient from each study group at (**A**) rest and (**B**) during regadenoson stress (at maximal vasodilation). The results of a patient who recently recovered from coronavirus disease (COVID-19) (red curve), a patient with hypertrophic cardiomyopathy (HCM) (black curve) and of a healthy control (blue curve) are shown in comparison.

Detailed data regarding VENC-based CSF measurements at rest and stress as well as respective ratios are outlined in Table 3. A slightly higher resting CSF was measured in COVID-19 patients compared to controls (1.78 [1.19–2.23] ml vs. 1.14 [0.91–1.32] ml, p = 0.048) regarding CSF per beat and also compared to HCM patients (1.07 [0.77–1.41] vs. 0.73 [0.44–0.91] ml/min/g, p = 0.016) regarding CSF per minute and gram heart muscle. The lowest stress-CSF values were measured in COVID-19 patients whereas the highest ones were obtained in controls (3.33 [2.76–4.20] ml/beat vs. 5.32 [3.66–5.52] ml/beat; p = 0.05). The resulting global MPR value was significantly lower in COVID-19 patients compared to controls (1.96 [1.51–2.78] per beat vs. 3.42 [2.90–5.45] per beat, p = 0.001 as well as 2.73 [2.10–4.15–11] per min vs. 4.82 [3.70–6.68] per min, p = 0.005). In spite of lower absolute values, no significant differences regarding MPR parameters were detected between COVID-19 patients and HCM patients. Representative CSF curves from patients of each group are demonstrated in Fig. 2.

**Table 3:** Coronary sinus flow (CSF) and global myocardial perfusion reserve (MPR) analysis.

	COVID-19	HCM	Control	p-value (COVID-19 vs. HCM)	p-value (COVID-19 vs. Control)
	N = 22	N = 16	N = 17		
CSF rest, ml/beat	1.78 (1.19–2.23)	1.35 (0.99–1.98)	1.14 (0.91–1.32)	0.27	**0.048**
CSF rest, ml/min	111 (73–153)	96 (65–131)	86 (66–111)	0.33	0.14
CSF rest, ml/min/g	1.07 (0.77–1.41)	0.73 (0.44–0.91)	0.84 (0.58–1.06)	**0.016**	0.16
CSF stress, ml/beat	3.33 (2.76–4.20)	3.47 (2.48–4.64)	5.32 (3.66–5.52)	1.00	0.05
CSF stress, ml/min	314 (221–457)	306 (160–395)	455 (380–507)	1.00	0.05
CSF stress, ml/min/g	3.23 (2.16–4.11)	2.33 (1.29–2.80)	3.90 (3.27–4.82)	0.06	0.24
MPR per beat	1.96 (1.51–2.78)	2.47 (1.42–3.54)	3.42 (2.90–5.45)	1.00	**0.001**
MPR per min	2.73 (2.10–4.15)	3.07 (1.73–4.86)	4.82 (3.70–6.68)	1.00	**0.005**

Bold values indicates p-value < 0.05.

**Figure 3:**
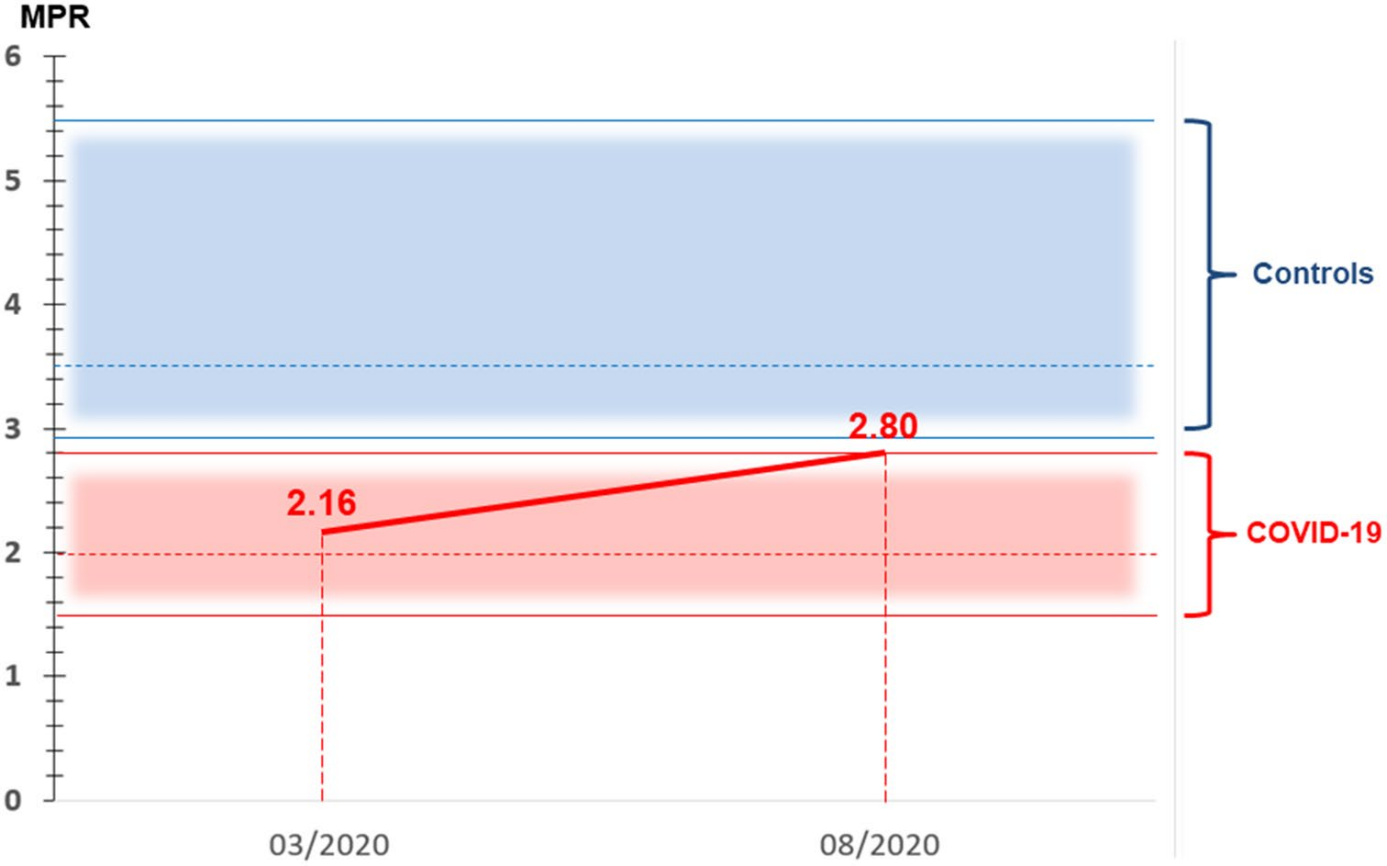
Graph demonstrating serial cardiovascular magnetic resonance (CMR) results of one COVID-19 patient with a repeated stress CMR study (as part of a separate study protocol) approximately five months after the first CMR study that was performed within the scope of the present study. In this patient, clinical symptoms of exertional dyspnea (initially NYHA III in March 2020) had slightly improved (NYHA II in August 2020). At the same time, an increase in global MPR from 2.16 (in March 2020) to 2.80 (in August 2020) was observed.

Finally, there was one COVID-19 patient with a repeated stress CMR study (as part of a separate study protocol) approximately five months after the first CMR study that was performed within the scope of the present study (Fig. 3). In this patient, clinical symptoms of exertional dyspnoea (initially NYHA III in March 2020) had slightly improved (NYHA II in August 2020). At the same time, an increase in global MPR from 2.16 (in March 2020) to 2.80 (in August 2020) was observed.

## Discussion

To the best of our knowledge, the present clinical study is the first one that assessed the presence of CMD in patients who recently recovered from COVID-19 using a non-invasive CMR-based approach. The major results of this hypothesis-generating pilot-study suggest that (a) CMD is present in (at least some) post-COVID-19 patients and (b) the presence of CMD in post-COVID-19 may contribute to the cause of ongoing exertional dyspnoea and PVFS in these patients.

Despite innumerable studies addressing the pathophysiology of COVID-19 infection that were published since the first COVID-19 outbreak in January 2020, the exact pathomechanism as well as the determinants of myocardial injury in COVID-19 are still not well understood^13^. Most conceptual models of COVID-19-associated myocardial injury address the acute disease phase only, and consider both direct endothelial injury and secondary (auto-)immune responses that cause a massive proinflammatory cytokine release as well as prothrombotic milieu^1,3,14^. Obviously, such pathophysiological changes during the acute phase may also have long-lasting effects on the coronary microvasculature, thereby further aggravating endothelial/myocardial injury and causing sustained CMD—even after resolution of acute inflammation (!). As outlined previously^15^, the (auto-)regulation and modulation of coronary blood flow in response to different stimuli (such as physical exercise) is disturbed in patients with CMD. Therefore, symptoms of (chronic) exertional dyspnoea may occur in those patients with CMD. Hence, we hypothesized that CMD is present in post-COVID-19 patients still suffering from exertional dyspnoea and PVFS and that CMR-based CSF measurements would allow to detect and prove the presence of CMD.

In the present pilot-study, we did not include severely diseased COVID-19 patients with critical illness and/or acute respiratory distress syndrome (ARDS) during the acute phase, but rather looked at those post-COVID-19 patients who did not require mechanical ventilatory or catecholaminergic support during the acute phase, but suffered from ongoing exertional dyspnoea and PVFS following the acute phase. Moreover, since the presence of CMD is well known in patients with classic HCM due to perivascular and interstitial fibrosis with adverse remodelling of coronary arterioles^8,16^, we compared our CSF-based measurements of MPR in post-COVID-19 patients not only to healthy controls but also to HCM patients. While global MPR was significantly reduced in post-COVID-19 patients compared to healthy controls, there was no substantial difference in global MPR between post-COVID-19 and HCM patients—suggesting a similar degree of CMD in both groups. In principle, the validated CSF-based measurement of MPR only allows for the assessment of “global” myocardial perfusion—but does not enable assessment of segmental differences. However, in case of systemic diseases such as COVID-19 a rather diffuse impairment of CMD is expected. Hence, our CSF-based measurement of “global” MPR should be highly appropriate within the context of the present study.

Recently, Rovas et al. performed intravital microscopy to quantify vascular density and glycocalyx dimensions in sublingual microvessels of hospitalized adult patients with moderate-to-severe or critical COVID-19^17^. Interestingly, COVID-19 patients showed an up to 90% reduction in vascular density, almost exclusively limited to small capillaries. In addition, several serum markers of endothelial dysfunction were increased and correlated with disease severity in COVID-19 patients. Hence, the authors concluded that COVID-19 is accompanied by endothelial activation, glycocalyx damage, and severe capillary impairment and that COVID-19 might have a distinctive vascular phenotype. Obviously, the exact pathomechanism leading to reduced global MPR in post-COVID-19 patients in the present study is unclear. However, based on studies like that of Rovas et al. and previous studies addressing coronary physiology in case of viral myocarditis^17–20^, we speculate that (a) endothelial dysfunction with impaired vasomotility and reduced vasodilatory capacity of coronary resistance vessels, (b) rarefaction of coronary arterioles and capillaries in the myocardium and (c) microvascular obstruction due to the preceding prothrombotic milieu may play a major role for the occurrence of CMD in post-COVID-19 patients.

As outlined in more detail previously^8^, the independent prognostic value of CSF-based MPR measurements was shown by several studies in the last years. For example, the prognostic value of CSF-based MPR to predict major adverse cardiac events (MACE) was evaluated in patients with CAD in a large prospective setting^10^. During a median follow-up time of 2.3 years, the CSF-based MPR showed a similar predictive value as the presence of > 10% ischemia in qualitative first-pass perfusion imaging in patients with known CAD and an even higher benefit in patients with suspected CAD (hazard ratios for MACE with CSF-based MPR compared to the presence of perfusion defects were 14.2 vs. 6.5). It needs to be considered that CSF-based MPR is not only reduced by the presence of obstructive epicardial stenosis—but is also affected by relevant microvascular changes. This notion is further supported by the fact that an incremental prognostic value of CMR-derived MPR was detected for patients with diabetes undergoing stress CMR imaging^21^. The annualized MACE rate was substantially higher in those patients with MPR < 2.0—regardless of the presence or absence of LGE. Whether a reduced MPR value (obtained from CSF measurements) will also have a “prognostic” value in post-COVID-19 patients, needs to be further analysed in future studies.

Finally, serial CMR data comprising serial MPR values from individual COVID-19 patients are highly desired in order to better assess the relationship and of course causality between clinical symptoms and underlying severity of CMD. Obviously, we cannot provide any MPR data from our COVID-19 patients regarding the MPR values prior to the respective COVID-19 infection. However, we were able to repeat our stress CMR protocol—including CSF measurements—in one COVID-19 patient five months after his first CMR study (Fig. 3). Concurrent to his clinical improvement, a substantial increase in global MPR was observed at least in this single patient—further supporting the relationship between CMR-based assessment of CMD and clinical symptoms of exertional dyspnoea. Obviously, more comprehensive imaging data—including strain data—will be helpful to improve our understanding of the underlying pathophysiology and the relationship of specific imaging parameters to clinical outcome^22^.

### Limitations

Although we did not include severely diseased COVID-19 patients with critical illness and/or ARDS during the acute phase and looked only at those post-COVID-19 patients who did not require mechanical ventilatory or catecholaminergic support during the acute phase, we cannot definitely rule out ongoing pulmonary disease and/or occult (e.g. subsegmental) pulmonary embolism as a confounding/additional cause of dyspnoea in some patients since data from computed tomography of the thorax were not available in all patients. Moreover, this was a hypothesis-generating pilot-study with a rather small sample size. Since post-COVID-19 patients, HCM patients and controls were not individually matched on age and sex, potential bias caused by a different age distribution and/or percentage of arterial hypertension cannot be excluded.

## Conclusion

In post-COVID-19 patients with persistent exertional dyspnoea and PVFS, a significantly reduced MPR suggestive of CMD—similar to HCM patients—was observed in the present study. A reduction in MPR can be caused by preceding SARS-CoV-2-associated direct as well as secondary triggered mechanisms leading to diffuse CMD, and may explain ongoing symptoms of exercise dyspnoea and PVFS in some patients after COVID-19 infection.

## Data Availability

The datasets used and/or analysed during the current study are available from the corresponding author on reasonable request.

## Abbreviations

CAD: Coronary artery disease
CFR: Coronary flow reserve
CMD: Coronary microvascular dysfunction
CMR: Cardiovascular magnetic resonance
COVID-19: Coronavirus disease 2019
CS: Coronary sinus
CSF: Coronary sinus flow
HCM: Hypertrophic cardiomyopathy
HF: Heart failure
IQR: Interquartile range
LGE: Late-gadolinium-enhancement
LV: Left ventricle
LV-EDV: Left ventricular end-diastolic volume
LV-EF: Left ventricular ejection fraction
LV-ESV: Left ventricular end-systolic volume
MPR: Myocardial perfusion reserve
PVFS: Post-viral fatigue syndrome
ROI: Region of interest
SARS-CoV-2: Severe acute respiratory syndrome coronavirus type 2
VENC: Velocity encoding

## References

[1] Bikdeli B, Madhavan MV, Jimenez D, Chuich T, Dreyfus I, Driggin E, Nigoghossian C, Ageno W, Madjid M, Guo Y, Tang LV, Hu Y, Giri J, Cushman M, Quere I, Dimakakos EP, Gibson CM, Lippi G, Favaloro EJ, Fareed J, Caprini JA, Tafur AJ, Burton JR, Francese DP, Wang EY, Falanga A, McLintock C, Hunt BJ, Spyropoulos AC, Barnes GD, Eikelboom JW, Weinberg I, Schulman S, Carrier M, Piazza G, Beckman JA, Steg PG, Stone GW, Rosenkranz S, Goldhaber SZ, Parikh SA, Monreal M, Krumholz HM, Konstantinides SV, Weitz JI, Lip GYH (2020). COVID-19 and thrombotic or thromboembolic disease: Implications for prevention, antithrombotic therapy, and follow-up: JACC state-of-the-art review. J. Am. Coll. Cardiol..

[2] Varga Z, Flammer AJ, Steiger P, Haberecker M, Andermatt R, Zinkernagel AS, Mehra MR, Schuepbach RA, Ruschitzka F, Moch H (2020). Endothelial cell infection and endotheliitis in COVID-19. Lancet.

[3] Ciceri F, Beretta L, Scandroglio AM, Colombo S, Landoni G, Ruggeri A, Peccatori J, D'Angelo A, De CF, Rovere-Querini P, Tresoldi M, Dagna L, Zangrillo A (2020). Microvascular COVID-19 lung vessels obstructive thromboinflammatory syndrome (MicroCLOTS): An atypical acute respiratory distress syndrome working hypothesis. Crit Care Resusc..

[4] Al-Ani F, Chehade S, Lazo-Langner A (2020). Thrombosis risk associated with COVID-19 infection A scoping review. Thromb. Res..

[5] Townsend L, Dyer AH, Jones K, Dunne J, Mooney A, Gaffney F, O’Connor L, Leavy D, O'Brien K, Dowds J, Sugrue JA, Hopkins D, Martin-Loeches I, Ni CC, Nadarajan P, McLaughlin AM, Bourke NM, Bergin C, O'Farrelly C, Bannan C, Conlon N (2020). Persistent fatigue following SARS-CoV-2 infection is common and independent of severity of initial infection. PLoS One.

[6] Bulfamante GP, Perrucci GL, Falleni M, Sommariva E, Tosi D, Martinelli C, Songia P, Poggio P, Carugo S, Pompilio G (2020). Evidence of SARS-CoV-2 transcriptional activity in cardiomyocytes of COVID-19 patients without clinical signs of cardiac involvement. Biomedicines.

[7] Koskenvuo JW, Sakuma H, Niemi P, Toikka JO, Knuuti J, Laine H, Komu M, Kormano M, Saraste M, Hartiala JJ (2001). Global myocardial blood flow and global flow reserve measurements by MRI and PET are comparable. J. Magn. Reson. Imaging.

[8] Bietenbeck M, Florian A, Shomanova Z, Meier C, Yilmaz A (2018). Reduced global myocardial perfusion reserve in DCM and HCM patients assessed by CMR-based velocity-encoded coronary sinus flow measurements and first-pass perfusion imaging. Clin. Res. Cardiol..

[9] Kato S, Saito N, Kirigaya H, Gyotoku D, Iinuma N, Kusakawa Y, Iguchi K, Nakachi T, Fukui K, Futaki M, Iwasawa T, Kimura K, Umemura S (2016). Impairment of coronary flow reserve evaluated by phase contrast cine-magnetic resonance imaging in patients with heart failure with preserved ejection fraction. J. Am. Heart Assoc..

[10] Kato S, Saito N, Nakachi T, Fukui K, Iwasawa T, Taguri M, Kosuge M, Kimura K (2017). Stress perfusion coronary flow reserve versus cardiac magnetic resonance for known or suspected CAD. J. Am. Coll. Cardiol..

[11] Schwitter J, DeMarco T, Kneifel S, von Schulthess GK, Jorg MC, Arheden H, Ruhm S, Stumpe K, Buck A, Parmley WW, Luscher TF, Higgins CB (2000). Magnetic resonance-based assessment of global coronary flow and flow reserve and its relation to left ventricular functional parameters: A comparison with positron emission tomography. Circulation.

[12] Shomanova Z, Florian A, Bietenbeck M, Waltenberger J, Sechtem U, Yilmaz A (2017). Diagnostic value of global myocardial perfusion reserve assessment based on coronary sinus flow measurements using cardiovascular magnetic resonance in addition to myocardial stress perfusion imaging. Eur. Heart J. Cardiovasc. Imaging.

[13] Saba L, Gerosa C, Fanni D, Marongiu F, La NG, Caocci G, Barcellona D, Balestrieri A, Coghe F, Orru G, Coni P, Piras M, Ledda F, Suri JS, Ronchi A, D'Andrea F, Cau R, Castagnola M, Faa G (2020). Molecular pathways triggered by COVID-19 in different organs: ACE2 receptor-expressing cells under attack? A review. Eur. Rev. Med. Pharmacol. Sci..

[14] Pellegrini D, Kawakami R, Guagliumi G, Sakamoto A, Kawai K, Gianatti A, Nasr A, Kutys R, Guo L, Cornelissen A, Faggi L, Mori M, Sato Y, Pescetelli I, Brivio M, Romero M, Virmani R, Finn AV (2021). Microthrombi as a major cause of cardiac injury in COVID-19: A pathologic study. Circulation.

[15] Yilmaz A, Sechtem U (2012). Angina pectoris in patients with normal coronary angiograms: Current pathophysiological concepts and therapeutic options. Heart.

[16] Crea F, Camici PG, Bairey Merz CN (2014). Coronary microvascular dysfunction: An update. Eur. Heart J..

[17] Rovas A, Osiaevi I, Buscher K, Sackarnd J, Tepasse PR, Fobker M, Kuhn J, Braune S, Gobel U, Tholking G, Groschel A, Pavenstadt H, Vink H, Kumpers P (2021). Microvascular dysfunction in COVID-19: The MYSTIC study. Angiogenesis.

[18] Guzik TJ, Mohiddin SA, Dimarco A, Patel V, Savvatis K, Marelli-Berg FM, Madhur MS, Tomaszewski M, Maffia P, D'Acquisto F, Nicklin SA, Marian AJ, Nosalski R, Murray EC, Guzik B, Berry C, Touyz RM, Kreutz R, Wang DW, Bhella D, Sagliocco O, Crea F, Thomson EC, McInnes IB (2020). COVID-19 and the cardiovascular system: Implications for risk assessment, diagnosis, and treatment options. Cardiovasc. Res..

[19] Vallbracht KB, Schwimmbeck PL, Kuhl U, Seeberg B, Schultheiss HP (2004). Endothelium-dependent flow-mediated vasodilation of systemic arteries is impaired in patients with myocardial virus persistence. Circulation.

[20] Yilmaz A, Mahrholdt H, Athanasiadis A, Vogelsberg H, Meinhardt G, Voehringer M, Kispert EM, Deluigi C, Baccouche H, Spodarev E, Klingel K, Kandolf R, Sechtem U (2008). Coronary vasospasm as the underlying cause for chest pain in patients with PVB19 myocarditis. Heart.

[21] Kato S, Fukui K, Kodama S, Azuma M, Iwasawa T, Kimura K, Tamura K, Utsunomiya D (2020). Incremental prognostic value of coronary flow reserve determined by phase-contrast cine cardiovascular magnetic resonance of the coronary sinus in patients with diabetes mellitus. J. Cardiovasc. Magn. Reson..

[22] Mishra AK, Lal A, Sahu KK, Kranis M, Sargent J (2020). Quantifying and reporting cardiac findings in imaging of COVID-19 patients. Monaldi Arch. Chest Dis..

